# A simulation study on matched case-control designs in the perspective of causal diagrams

**DOI:** 10.1186/s12874-016-0206-3

**Published:** 2016-08-20

**Authors:** Hongkai Li, Zhongshang Yuan, Ping Su, Tingting Wang, Yuanyuan Yu, Xiaoru Sun, Fuzhong Xue

**Affiliations:** Department of Biostatistics, School of Public Health, Shandong University, Jinan City, Shandong Province People’s Republic of China

**Keywords:** Simulation study, Matched case-control designs, Causal diagrams

## Abstract

**Background:**

In observational studies, matched case-control designs are routinely conducted to improve study precision. How to select covariates for match or adjustment, however, is still a great challenge for estimating causal effect between the exposure E and outcome D.

**Methods:**

From the perspective of causal diagrams, 9 scenarios of causal relationships among exposure (E), outcome (D) and their related covariates (C) were investigated. Further various simulation strategies were performed to explore whether match or adjustment should be adopted. The “*do calculus”* and “*back-door criterion*” were used to calculate the true causal effect (*β*) of E on D on the log-odds ratio scale. 1:1 matching method was used to create matched case-control data, and the conditional or unconditional logistic regression was utilized to get the estimators ($\overset{\frown }{\beta }$) of causal effect. The bias ($\overset{\frown }{\beta}\hbox{-} \beta$) and standard error ($SE\left(\overset{\frown }{\beta}\right)$) were used to evaluate their performances.

**Results:**

When C is exactly a confounder for E and D, matching on it did not illustrate distinct improvement in the precision; the benefit of match was to greatly reduce the bias for *β* though failed to completely remove the bias; further adjustment for C in matched case-control designs is still essential. When C is associated with E or D, but not a confounder, including an independent cause of D, a cause of E but has no direct causal effect on D, a collider of E and D, an effect of exposure E, a mediator of causal path from E to D, arbitrary match or adjustment of this kind of plausible confounders C will create unexpected bias. When C is not a confounder but an effect of D, match or adjustment is unnecessary. Specifically, when C is an instrumental variable, match or adjustment could not reduce the bias due to existence of unobserved confounders U.

**Conclusions:**

Arbitrary match or adjustment of the plausible confounder C is very dangerous before figuring out the possible causal relationships among E, D and their related covariates.

## Background

In observational studies, confounding factors (C) that are pre-exposure variables associated with the exposure E and the outcome D will distort the estimation of the target causal effect [1–4]. Generally, the magnitude of confounding bias mainly depends on the strength of the effects from confounder C to exposure E and from confounder C to outcome D. If one of these two effects is precisely null, confounding bias does not exist at all. Furthermore, the directions of effect from C to E and from C to D determine the direction of the bias. Usually, confounding factors could mainly lead to three kinds of biases in an attempt to find the causal effect from E to D, including over-estimation, under-estimation, or even missing the direction of the effect [5].

In analytic epidemiology, various strategies could be adopted to remove confounding bias, such as Restriction, Adjustment, Stratification [6, 7], while strategy of matching on confounders C (e.g. matched case-control designs) mainly focuses on improving estimation precision of the effect of E on D, rather than removing confounding bias [8, 9]. For matched case-control designs, matching refers to the selection of controls group that is identical, or nearly so, to the cases group with respect to the distribution of one or more potentially confounding factors. Generally, two matching strategies, including individual matching and frequency matching, could be selected to force the distribution of the matching factors to be identical across groups of individuals [10]. In particular, individual matching involves selection of one or more controls group with matching factor values equal to cases group. From the perspective of causal diagrams, several qualitative studies had suggested that matching on confounders not only fails to remove confounding bias but also adds colliding bias [11–15]. Therefore, it is still necessary to adjust for the matching variables.

However, for obtaining unbiased and precise estimation, it is crucial to choose matching variables correctly and further determine whether they should be adjusted for. For matching variables, matching on common child nodes of exposure and outcome, or mediators of the exposure and outcome will generally lead to irremediable bias [13, 14]. For further adjustment, conditional logistic regression models are customarily used to adjust for matching variables, which just provide conditional rather than causal estimation of odds ratio [16]. Sometimes, unconditional logistic regression models can also be adopted to adjust for matching variables, but they will lead to lower precision for the parameters estimation when the number of matched variables is larger under given limited sample size [17].

**Fig. 1:**
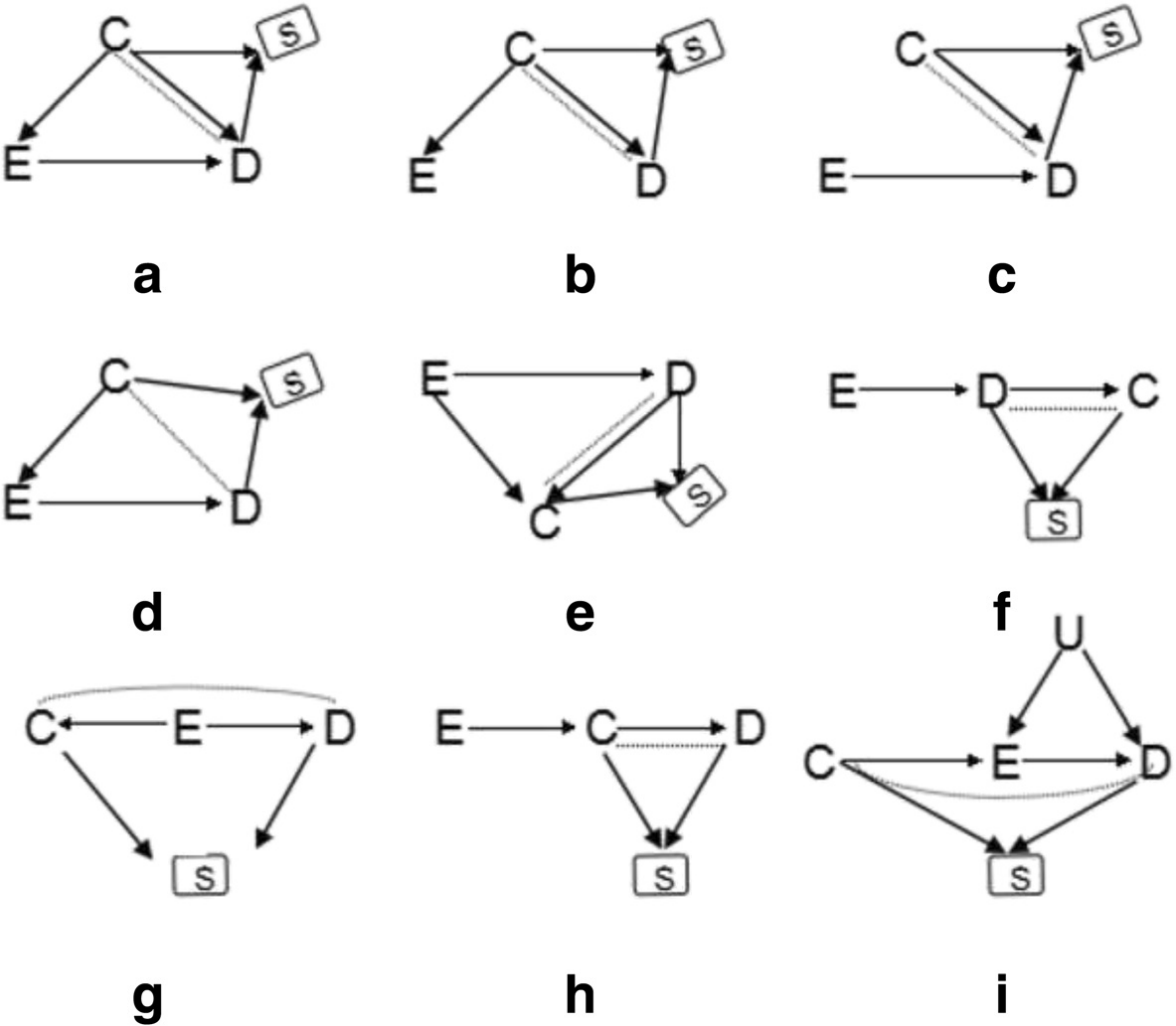
Nine simulation scenarios. *E, C, D* indicate exposure, matching factor, outcome, respectively. Let variable S indicate whether a person is selected for case-control study or not, the *square* around S indicates the analysis is conditional on individuals having been selected into the matched case-control study. *Dashed line* C--D show the colliding bias (i.e., selective bias) due to matching on C. S is a collider on C→S←D. Colliding bias will arise if conditioning on colliding node (i.e., S). **a**) C is a confounder for the exposure E and the outcome D; **b**) C is a common cause of E and D with an absence of cause effect between them; **c**) C is an independent cause of D; **d**) C is a cause of E, but has no direct causal effect on D; **e**) C is a common effect (i.e. collider) of E and D; **f**) C is an effect of outcome D; **g**) C is an effect of exposure E; **h**) C is a mediator of causal path from E to D; **i**) C is an instrumental variable for E and D

In this paper, we performed various quantitative simulations under the following 9 scenarios to illustrate the benefits of correct match and further proper adjustment, and to highlight the consequences of improper match and further inappropriate adjustment. a) C is a confounder for the exposure E and the outcome D (Fig. 1a); b) C is a common cause of E and D with an absence of cause effect between them (Fig. 1b); c) C is an independent cause of D (Fig. 1c); d) C is a cause of E, but has no direct causal effect on D (Fig. 1d); e) C is a common effect (i.e. collider) of E and D (Fig. 1e); f) C is an effect of outcome D (Fig. 1f); g) C is an effect of exposure E (Fig. 1g); h) C is a mediator of causal path from E to D (Fig. 1h); i) C is an instrumental variable for E and D (Fig. 1i). All above scenarios almost involve common roles of C in analytic epidemiology.

## Methods

### A brief introduction to causal diagrams and calculation of causal effect

In the past few decades, causal diagrams, one kind of directed acyclic graphs (DAGs), have been widely used to visually summarize hypothetical causal relations among variables of interest. Modern causal diagrams were more recently developed to merger probability theory with path diagrams [2, 18–20]. The resulting theory provides a powerful yet intuitive device for deducing the statistical associations implied by causal relations. Furthermore, given a set of observed statistical associations, a researcher armed with causal diagrams theory can systematically characterize all causal analysis. In causal diagrams, the *d-Separation* criterion is an essential graphic rule for linking causal relations to statistical associations [20, 21]. They help epidemiologists to draw logically sound conclusions about certain types of statistical relations and facilitate many tasks, such as understanding confounding bias and selection bias [15], choosing covariates for adjustment or match [10], analyzing direct and indirect effects [22], using instrumental variable to estimate causal effect when unobserved confounders exist [23]. In this paper, we used causal diagrams to illustrate the relationships among variables in above 9 scenarios.

Furthermore, *do-calculus* together with *back-door criterion* proposed by Pearl [20, 24, 25] were used to calculate the causal effect of exposure (X) on outcome (Y). Given a causal diagram *G*, together with non-experimental data on a subset *V* of observed variables in *G*, we estimate the causal effect of X on Y by calculating *P*(*y*|d*o*(*X* = *x*)) from a sample estimation of *P*(V = *v*). Namely, we aim to estimate what the intervention *do*(X = x) would have on a set of response variable *Y*, where *X* and *Y* are two subsets of *V*. For identifying *P*(*y*|d*o*(*X* = *x*)), the “*back-door criterion*” [20] was further used to test if a set *Z* ⊆ *V* of variables is sufficient, where Z satisfied the following conditions. (i) it blocked every path from X to Y that has an arrow into X (“blocks the back door”); and (ii) no node in Z is a descendant of X. If a set of variable Z satisfies the back-door criterion relative to (X, Y), then the causal effect of X on Y is identifiable and is calculated by the following formula,

$$ P\left(y\Big| do\left(X=x\right)\right)={\displaystyle \sum_ZP\left(y\Big|x,z\right)P(z)} $$

In this paper, this formula was used to calculate the true causal effect *β* of exposure E on outcome D from source population. It was regarded as a gold standard to assess the bias of estimation in all 9 simulation scenarios.

### Simulation scenarios

Figure 1 showed the causal diagrams of 9 simulation scenarios for estimating causal effect of E on D, which illustrated 9 different roles of C respectively. Based on Fig. 1(a) to (i), Monte Carlo simulations were used to generate simulation data. We made the following assumptions for the simulation: 1) all variables are binary following a Bernoulli distribution; 2) the correlations between variables are positive unless otherwise specified; and 3) the association between covariates (E and C) and the outcome D is log-linearly additive effect. Logistic regression models were used to simulate child nodes from their corresponding parent nodes. Take scenario 1 [seeing Fig. 1(a)] as an example, let *P*(*C* = 1) = *π***,** then *P*(*E* = 1|*C*) = exp(*α*_0_ + *α*_1_*C*)/[1 + exp(*α*_0_ + *α*_1_*C*)] for the child node E from its parent node C; similarly, *P*(*D* = 1|*C*, *E*) = exp(*β*_0_ + *β*_1_*C* + *β*_2_*E*)/[1 + exp(*β*_0_ + *β*_1_*C* + *β*_2_*E*)]; where the parameters *α*_0_, *β*_0_ denoted the baseline prevalence of E and D respectively, and each effect parameter (*α*_1_, *β*_1_, *β*_2_) refers to the log-odds ratio conditional on other covariates. The simulated source population with 100,000 subjects was generated from above procedure. 1000 cases were randomly sampled from this simulated source population with D = 1, while 1000 controls were randomly sampled from D = 0; so far none-matched case-control data with 1000 cases and 1000 controls was created. For matched case-control data, we still used the above same 1000 cases as the cases group, for individual with C = 1 in cases group, we matched its control by randomly sampling a subject with C = 1 and D = 0 from the source population; similarly, for individual with C = 0, we matched its control with C = 0 and D = 0 from the source population.

Besides, unconditional and conditional regression models were applied to above two datasets to assess their performances. For non-matched case-control data, both unconditional logistic regression model without adjusting for C, $\log it\left(p\left(D=1\Big|E\right)\right)={\beta}_0+{\beta}_{{}_1}^{\prime}\mathrm{E}$, (model 1), and with adjusting for C, log *it*(*p*(*D* = 1|*E*, *C*)) = *β*_0_ + *β*_1_^″^E + *β*_2_C, (model 2), were performed for comparing their bias (${\overset{\frown }{\beta}}_1\hbox{-} \beta$, where ${\overset{\frown }{\beta}}_1$ was the estimation by the logistic regression models, while *β* was the true causal effect from source population) and precision by the standard error of ${\overset{\frown }{\beta}}_1$ ($\mathrm{S}\mathrm{E}\left({\overset{\frown }{\beta}}_1\right)$). For matched case-control data, the following three models were used to compare their bias (${\overset{\frown }{\beta}}_1\hbox{-} \beta$) and precision ($\mathrm{S}\mathrm{E}\left({\overset{\frown }{\beta}}_1\right)$): model 3) unconditional logistic regression without adjusting for C; model 4) unconditional logistic regression with adjusting for C; and model 5) conditional logistic regression.

Various simulation scenarios were performed by varying across a target effect parameter [e.g. C → E in Fig. 1(a)] and keeping all others constant to explore the trends of bias (${\overset{\frown }{\beta}}_1\hbox{-} \beta$) and standard error ($\mathrm{S}\mathrm{E}\left({\overset{\frown }{\beta}}_1\right)$). 1000 simulations were repeated in each scenario. All simulation studies were conducted using software R from CRAN (http://cran.r-project.org/).

## Results

### Scenario 1 (C is a confounder for E and D, Fig. 1a)

Theoretically, in this scenario, the confounder C is *d-connected* with outcome D via two natural paths: C → D and C → E → D, which contribute to the crude association between C and D. Nevertheless, under matched case-control designs, C is unconditionally independent of D due to the identical distribution of C in cases and controls group (i.e. the sum of C → D, C → E → D and C--D is null). Furthermore, the path C--D is of equal magnitude, but opposite direction to the C → E → D and C → D. Therefore, the joint distribution of E, C and D is unfaithful to the DAG of Fig. 1a due to matching on C. As C is a confounder, both paths C → E and C → D will lead to the bias for E on D before matching, while after matching, a new colliding bias path C--D is created and the two bias paths (C → E, C → D) still exist. In this situation, the total bias is contributed by the path of C → E, C → D and C--D [13–15].

**Fig. 2:**
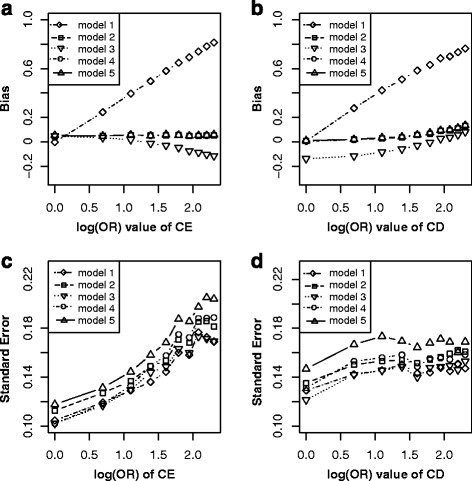
Bias (*upper panels*) and standard error (i.e. SE, *lower panels*) of log transformed odds ratio estimations for different effect sizes of CE and CD. Each *line* indicated one model. The *left panel* displayed the bias and standard error on the estimated values of exposure E for different odds ratio (from 1 to 10) of CE respectively. The *right panel* showed the bias and standard error of estimated values on exposure E for different odds ratio (from 1 to 10) of CD respectively. *Note*: model 1, unconditional logistic regression model without adjusting for C for non-matched case-control designs; model 2, unconditional logistic regression model with adjusting for C for non-matched case-control designs; model 3, unconditional logistic regression model without adjusting for C for matched case-control designs; model 4, unconditional logistic regression model with adjusting for C for matched case-control designs; model 5, conditional logistic regression model with adjusting for C for matched case-control designs

Figure 2 showed the simulation results under scenario 1. It indicated that given other parameters fixed and varying across the effect of C → E (Fig. 2a), the bias (${\overset{\frown }{\beta}}_1\hbox{-} \beta$) elevated linearly with effects of C → E increasing in the model without adjusting for C under non-matched case-control designs (model 1), while elevated in the opposite direction with effects of C → E increasing in the model without adjusting for C under matched case-control designs (model 3); after adjusting for C, the bias was approximate to zero in all models of adjustment for C under non-matched case-control designs (model 2) and matched case-control designs (model 4 or model 5). For their precision (Fig. 2c), the $SE\left({\overset{\frown }{\beta}}_1\right)$ of all above five models increased with effects of C → E increasing, and model 5 obtained largest standard error, followed by model 4, model 2, model 3, model 1. Similarly, given other parameters fixed and varying across C → D (Fig. 2b), the bias (${\overset{\frown }{\beta}}_1\hbox{-} \beta$) still elevated linearly with effects of C → D increasing in model 1, while lowered with effects of C → D increasing in model 3. After adjusting for C, the bias was still nearly approximate to zero in model 2, model 4 or model 5. For their precision (Fig. 2d), the $SE\left({\overset{\frown }{\beta}}_1\right)$ of all above five models kept stable with effects of C → D increasing, and model 5 attained largest standard error, followed by model 4, model 2, model 1, model 3. These results suggested that confounding bias and colliding bias generally changed in opposite directions and adjustment was indispensable after matching on C.

### Scenario 2 (C is a common cause of exposure E and outcome D without causal effect between them, Fig. 1b)

It is similar to scenario 1 (Fig. 1a) except that instead of having causal effect between E and D. In this situation, the path C → D leads to the association of C and D in a non-matched case-control designs. But two effect paths of C and D offset each other after matching, that is the effect of C--D is of equal magnitude, but opposite direction to C → D [14].

**Fig. 3:**
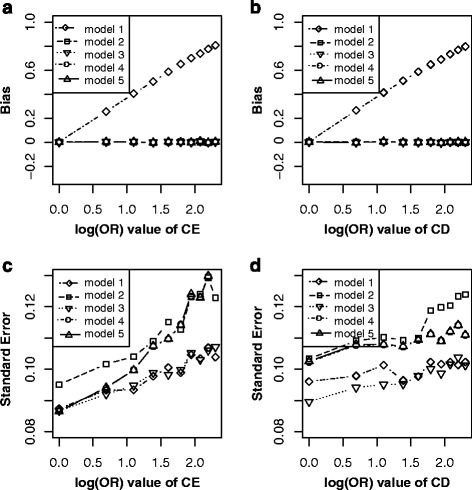
Bias (*upper panels*) and standard error (i.e. SE, *lower panels*) of log transformed odds ratio estimations for different effect sizes of CE and CD. Each *line* indicated one model. The *left panel* displayed the bias and standard error of different odds ratio (from 1 to 10) of CE. The *right panel* showed the bias and standard error of different odds ratio (from 1 to 10) of CD. *Note*: model 1, unconditional logistic regression model without adjusting for C for non-matched case-control designs; model 2, unconditional logistic regression model with adjusting for C for non-matched case-control designs; model 3, unconditional logistic regression model without adjusting for C for matched case-control designs; model 4, unconditional logistic regression model with adjusting for C for matched case-control designs; model 5, conditional logistic regression model with adjusting for C for matched case-control designs

Simulation showed that (Fig. 3): keeping other parameters constant, and varying across C → E (Fig. 3a), the bias (${\overset{\frown }{\beta}}_1\hbox{-} \beta$) elevated linearly with effects of C → E increasing in the model without adjusting for C under non-matched case-control designs (model 1), while approximate unbiased estimations were got in model 2, model 3, model 4 and model 5. All five models revealed an increased effect with effects of C → E increasing. Among them, model 2 got largest standard error, followed by model 4, model 5, model 3 and model 1. Similarly, as E ← C → D is a confounding path (Fig. 3b), the bias (${\overset{\frown }{\beta}}_1\hbox{-} \beta$) elevated linearly with effects of C → D increasing in model 1, while the bias was almost null after adjustment (model 2, model 3, model 5) or match (model 4). The $SE\left({\overset{\frown }{\beta}}_1\right)$ revealed a linearly increasing trend for the five models, while the model 2 illustrated largest standard error, followed by model 4, model 5, model 1, model 3. These results indicated that both matching and adjustment could block the bias path E ← C → D, but adjustment for C would lead to lower precision. Therefore, the best choice is the model without adjusting for C in matched case-control designs (model 3) in scenario 2.

### Scenario 3 (C is a cause of outcome D, Fig. 1c)

As C is not a confounder, C and E are independent causes of D, respectively, the marginal effect from E to D is an unbiased estimator. In this situation, matching on or adjustment for C will inevitably lead to bias for E on D due to conditional on C by matched case-control designs or logistic regression model [14].

**Fig. 4:**
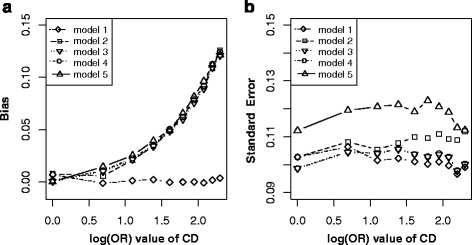
Bias (*left panels*) and standard error (i.e. SE, *right panels*) of log transformed odds ratio estimations for different effect sizes of CD. Each *line* indicated one model. *Note*: model 1, unconditional logistic regression model without adjusting for C for non-matched case-control designs; model 2, unconditional logistic regression model with adjusting for C for non-matched case-control designs; model 3, unconditional logistic regression model without adjusting for C for matched case-control designs; model 4, unconditional logistic regression model with adjusting for C for matched case-control designs; model 5, conditional logistic regression model with adjusting for C for matched case-control designs

As expected, only model without adjusting for C in non-matched case-control designs (model 1) got unbiased and precise estimation (Fig. 4), and both match and adjustment would increase bias and lower precision with effects of C → D increasing in model 2 to model 5.

### Scenario 4 (C is a cause of exposure E, Fig. 1d)

**Fig. 5:**
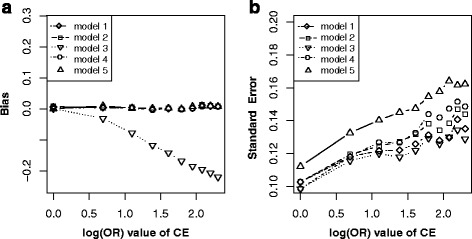
Bias (*left panels*) and standard error (i.e. SE, *right panels*) of log transformed odds ratio estimations for different effect sizes of CE. Each line represented one model. *Note*: model 1, unconditional logistic regression model without adjusting for C for non-matched case-control designs; model 2, unconditional logistic regression model with adjusting for C for non-matched case-control designs; model 3, unconditional logistic regression model without adjusting for C for matched case-control designs; model 4, unconditional logistic regression model with adjusting for C for matched case-control designs; model 5, conditional logistic regression model with adjusting for C for matched case-control designs

The C has a direct effect on E and an indirect effect on D through E. So C is not a confounder for E and D. In this situation, if matching on C, a new association is generated between C and D (denoted with C--D). Thus E ← C--D becomes an open bias path for E on D [14]. Simulation results (Fig. 5) supported above deductions, and revealed that only model without adjusting for C in matched case-control designs (model 3) led to bias (Fig. 5a). In matched case-control designs, although the bias could be remedied by adjusting for C, the precision (Fig. 5b) would become lower (model 4 and model 5).

### Scenario 5 (C is a common effect of exposure E and outcome D, Fig. 1e)

**Fig. 6:**
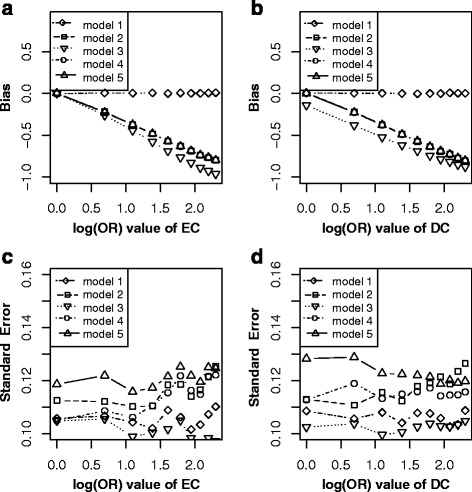
Bias (*left panels*) and standard error (i.e. SE, *right panels*) of log transformed odds ratio estimations for different effect sizes of EC and DC. Each *line* indicated one model. *Note*: model 1, unconditional logistic regression model without adjusting for C for non-matched case-control designs; model 2, unconditional logistic regression model with adjusting for C for non-matched case-control designs; model 3, unconditional logistic regression model without adjusting for C for matched case-control designs; model 4, unconditional logistic regression model with adjusting for C for matched case-control designs; model 5, conditional logistic regression model with adjusting for C for matched case-control designs

In this scenario, as C is not a confounder but a collider, match on or adjustment for C (model 2 to model 5) will generate colliding bias [14, 15]. The simulation results under varying across the effects of E → C and C ← D (Fig. 6) verified that only model without adjusting for C in non-matched case-control designs (model 1) got unbiased estimation.

### Scenario 6 (C is an effect of outcome D, Fig. 1f)

**Fig. 7:**
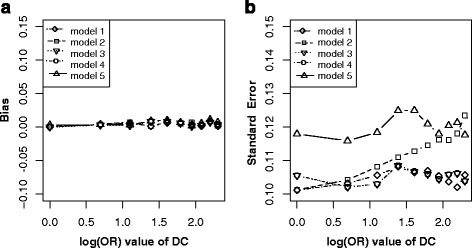
Bias (*left panels*) and standard error (i.e. SE, *right panels*) of log transformed odds ratio estimations for different effect sizes of DC. Each *line* indicated one model. *Note*: model 1, unconditional logistic regression model without adjusting for C for non-matched case-control designs; model 2, unconditional logistic regression model with adjusting for C for non-matched case-control designs; model 3, unconditional logistic regression model without adjusting for C for matched case-control designs; model 4, unconditional logistic regression model with adjusting for C for matched case-control designs; model 5, conditional logistic regression model with adjusting for C for matched case-control designs

In this scenario, the C is not a confounder but an effect (child node) of outcome D, so match on or adjustment for C is not necessary. Simulation results showed that both matching on C and adjusting for C did not lead to bias of *β* (Fig. 7a), but adjustment for C (model 2, model 4 and model 5) led to lower precision (Fig. 7b).

### Scenario 7 (C is an effect of exposure E, Fig. 1g)

**Fig. 8:**
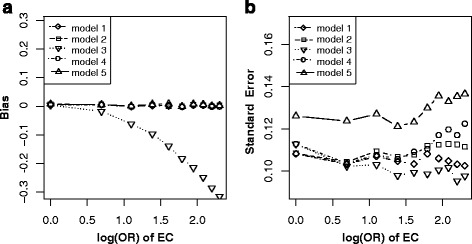
Bias (*left panels*) and standard error (i.e. SE, *right panels*) of log transformed odds ratio estimations for different effect sizes of EC. Each *line* indicated one model. *Note*: model 1, unconditional logistic regression model without adjusting for C for non-matched case-control designs; model 2, unconditional logistic regression model with adjusting for C for non-matched case-control designs; model 3, unconditional logistic regression model without adjusting for C for matched case-control designs; model 4, unconditional logistic regression model with adjusting for C for matched case-control designs; model 5, conditional logistic regression model with adjusting for C for matched case-control designs

For this scenario, although C is associated with E (E → C) and D (C ← E → D), it is not a confounder. In practice, it is difficult to distinguish it from confounder by statistical association study. Theoretically, matching on this kind of spurious confounders will open bias path E → C--D and thus lead to biased estimation of *β*. On the other hand, adjustment for C will not lead to biased estimation of *β* but will lower its precision. Simulation results are concordant with above deductions, which revealed the biased estimation of *β* (Fig. 8a) by matching on C (model 3), and showed lower precision (Fig. 8b) by adjusting for C (model 2, model 4 and model 5).

### Scenario 8 (C is a mediator of causal path from E to D, Fig. 1h)

**Fig. 9:**
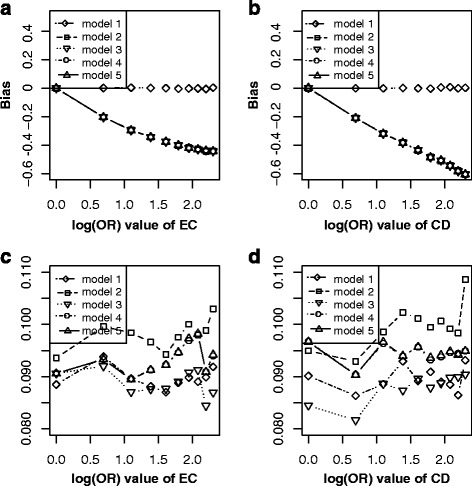
Bias (*upper panels*) and standard error (i.e. SE, *lower panels*) of log transformed odds ratio estimations for different effect sizes of EC and CD. Each *line* indicated one model. *Note*: model 1, unconditional logistic regression model without adjusting for C for non-matched case-control designs; model 2, unconditional logistic regression model with adjusting for C for non-matched case-control designs; model 3, unconditional logistic regression model without adjusting for C for matched case-control designs; model 4, unconditional logistic regression model with adjusting for C for matched case-control designs; model 5, conditional logistic regression model with adjusting for C for matched case-control designs

In this scenario, although C is associated with E (E → C) and D (C → D), it is not a confounder but a mediator. Matching on C will block the path E → C, while adjusting for C will block the path C → D. Therefore, either match or adjustment will inevitably block the causal path E → C → D, and thus leads to the biased estimation of *β* [14]. Both Fig. 9a and b illustrated that only model without adjusting for C in non-matched case-control designs (model 1) got unbiased estimation of *β* in the situation of varying across effects of E → C and C → D. In these two situations, lower precision of ${\overset{\frown }{\beta}}_1$ (Fig. 9c and d) were observed by adjusting for C (model 2, model 4 and model 5).

### Scenario 9 (C is an instrumental variable for E and D, Fig. 1i)

**Fig. 10:**
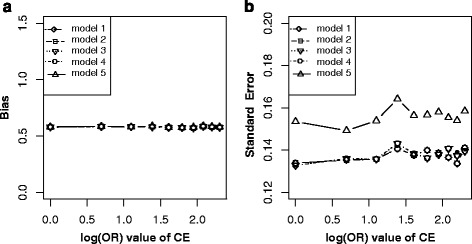
Bias (*left panels*) and standard error (i.e. SE, *right panels*) of log transformed odds ratio estimations for different effect sizes of CE. Each *line* indicated one model. *Note*: model 1, unconditional logistic regression model without adjusting for C for non-matched case-control designs; model 2, unconditional logistic regression model with adjusting for C for non-matched case-control designs; model 3, unconditional logistic regression model without adjusting for C for matched case-control designs; model 4, unconditional logistic regression model with adjusting for C for matched case-control designs; model 5, conditional logistic regression model with adjusting for C for matched case-control designs

In Fig. 10, we can easily find that C is not a confounder but an instrumental variable (IV), though C is associated with E (C → E) and D (C → E → D). This instrumental variable C can be used to control for the unobserved confounder U for estimating the causal effect of E on D [26]. However, instead of controlling for the confounding effect of U through either matching on or adjusting for C, the biased estimation for effect of E → D could not be reduced. The simulation results (Fig. 10) indicated that all the five models led to similar bias.

## Discussion

From the perspective of causal diagrams, several studies had claimed that matching on confounders C in matched case-control designs can improve estimation precision for the effect of exposure (E) on outcome (D), though it fails to remove confounding effect of C [8, 9]. Therefore, further adjustment for C using conditional or unconditional logistic regression model after matching is widely used to eliminate the confounding bias of C in analytic epidemiology [13, 14]. When C is exactly a confounder for E and D (scenario 1, Fig. 1a), however, our simulation results did not illustrate distinct improvement of precision for estimating effect of E on D by matching on C (model 3) comparing with by non-matching designs (model 1). Nevertheless, the benefit of matching on C was to greatly reduce the bias for estimating the effect of E on D (model 3) though failed to completely remove the bias (Fig. 2a and b). Further adjusting for C using logistic regression model (model 4 or model 5) after matching almost removed the bias (Fig. 2a and b). Our simulation results suggested that further adjusting for C in matched case-control designs is still essential, while adjustment (Fig. 2c and d) by unconditional logistic regression model (model 4) tend to be more precise than by conditional logistic regression (model 5). Similarly, in scenario 2 (Fig. 1b), C also is a confounder though the causal effect from E to D does not exist. In this situation, both matching on or adjusting for C could obtain unbiased estimation of E on D (Fig. 3), but matched case-control designs without adjusting for C (model 3) was the optimal strategy.

In practice, it is usually difficult to identify confounders just from statistical association [7, 27]. 1) In scenario 3 (Fig. 1c), both C and E are independent causes of D, matching on or adjustment for C will inevitably lead to bias for E on D due to conditional on C (Fig. 4) [14]. 2) In scenario 4 (Fig. 1d), C is associated with E (C → E) and D (C → E → D), but not a confounder. In this situation, matching on C, a new association was generated between C and D (denoted with C--D). Thus E ← C--D became an open bias path for E on D, and generated its biased estimation (Fig. 5). Fortunately, further adjustment for C after match could remedy this bias (model 4 and model 5 in Fig. 5) [14]. 3) In scenario 5 (Fig. 1e), C is not a confounder but a collider, match on or adjustment for C (model 2 to model 5) will inevitably generate colliding bias; only non-matched case-control designs without adjusting for C (model 1) got unbiased estimation (Fig. 6a and b) [14, 15]. 4) In scenario 8 (Fig. 1h), C is associated with E (E → C) and D (C → D), it is not a confounder but a mediator. Matching on C will block the path E → C, while adjusting for C will block the path C → D [14]. Therefore, either match or adjustment will inevitably block the causal path E → C → D, and thus lead to the biased estimation of *β* (Fig. 9). In this situation, only model without adjusting for C in non-matched case-control designs (model 1) got unbiased estimation of *β*. However, adjustment for C (model 2, model 4 and model 5) would reduce the precision of ${\overset{\frown }{\beta}}_1$ (Fig. 9c and d). It was, therefore, dangerous and improper to arbitrarily match on or adjust for the plausible confounder C [28].

Above simulation scenarios (scenario 1, 2, 3, 4, 5, 8) have been explored by shahar and Mansournia et al., but beyond that we proposed three new causal diagrams (scenario 6, 7, 9) with respect to match or adjustment strategies. Our simulation results showed that, for case-control study designs, when C is not a confounder but an effect (child node) of outcome D (scenario 6, Fig. 1f), match on or adjustment for C is not necessary (Fig. 7) in that it did not lead to biased estimation of *β* (Fig. 7a). In scenario 7 (Fig. 1g), C is associated with E (E → C) and D (C ← E → D), but not a confounder. Matching on this kind of spurious confounders would open bias path E → C--D and thus led to biased estimation of *β* (Fig. 8). Although adjusting for C did not lead to biased estimation of *β*, it would reduce precision (Fig. 8). Specifically, when C is an instrumental variable for E and D, although it is associated with E (C → E) and D (C → E → D), matching on or adjusting for it, the biased for effect of E → D could not be reduced (Fig. 10).

## Conclusions

In conclusion, for using match or adjustment strategy in case-control studies, investigators should firstly attempt to figure out the possible causal relationships among exposure (E), outcome (D) and their related covariates (C) empirically based on the etiologic and pathological mechanism and then determine whether match or adjustment should be adopted. Otherwise, arbitrary matching on or adjusting for the plausible confounder C is dangerous.

## Abbreviations

DAGs, directed acyclic graphs; IV, instrumental variable; SE, standard error
